# Pigmented Villonodular Synovitis Mimicking an Acute Septic Hip in an Eight-Year-Old Male

**DOI:** 10.7759/cureus.9895

**Published:** 2020-08-20

**Authors:** Anderson S Marshall, Adolfo L Molina, Daniel Reiff, Ryan Sheets, Michael Conklin

**Affiliations:** 1 Internal Medicine, University of Alabama at Birmingham, Birmingham, USA; 2 Pediatrics, Children's of Alabama, Birmingham, USA; 3 Pediatric Hospital Medicine, University of Alabama at Birmingham, Children's of Alabama, Birmingham, USA; 4 Pediatric Rheumatology, University of Alabama at Birmingham, Children's of Alabama, Birmingham, USA; 5 Internal Medicine/Pediatrics, University of Alabama at Birmingham, Birmingham, USA; 6 Orthopaedic Surgery, University of Alabama at Birmingham, Birmingham, USA

**Keywords:** pigmented villonodular synovitis, acute joint pain, pediatric orthopedic surgery, septic arthritis, pediatrics, hip mass, tenosynovial giant cell tumor

## Abstract

Pigmented villonodular synovitis (PVNS) is a less common but known cause of joint pain in the adult population. PVNS in pediatric patients is even more rare, with only case reports of occurrence in persons under the age of 18 years. Presentation is typically that of more insidious pain and limited range of motion, and is primarily seen in the knee joint. Diagnosis can be suspected with imaging, but ultimately surgical intervention is needed for tissue confirmation. We present a case of PVNS in a pediatric patient with acute symptoms concerning for a septic joint. The patient’s workup revealed a large effusion on hip ultrasound, with operative intervention pursued and further imaging deferred given the patient's symptom burden. A 4 × 1 × 1.5 cm intra-articular pigmented mass excised from the synovium in the operating room. The patient’s symptoms improved after the procedure, with pathology showing sheets of plump mononuclear cells in a collagenized stroma with hemosiderin deposits, confirming the diagnosis. This case highlights the importance of keeping non-infectious etiologies in the differential diagnosis of acute onset joint pain.

## Introduction

Acute joint pain is a complaint that is common place in the pediatric population. Often in the acute setting the focus is differentiating between an infectious process and an inflammatory one. A common workup includes checking laboratory markers of inflammation, multimodal imaging such as ultrasound and MRI, and a physical exam focused on the joint of concern. While infection is the most concerning and thought of cause of severe joint pain, atypical causes should be considered in a clinical context that does not collaborate with the patient's history. Pigmented villonodular synovitis (PVNS), also known as tenosynovial giant cell tumor, has a varying reported incidence most commonly cited at two new cases per million annually. Typical presentation is in the second to third decade of life. Pain, limited range of motion, and joint swelling/fullness are most often reported. This case is unique because of the acuity of our patient’s onset of symptoms and their rapid progression. It also identifies a possible genetic predisposition towards this diagnosis, as the patient’s father reported a similarly resected mass from his hip during adolescence.

## Case presentation

An eight-year-old African-American male presented as a transfer to the emergency department with a chief complaint of left hip pain. His symptoms began three days prior to presentation after getting hit with a dodgeball in the thigh or hip, rapidly progressing to inability to bear weight two days prior. No other trauma was reported. Aside from subjective swelling of the hip, no other infectious symptoms were reported. Past histories were relevant for attention deficit hyperactivity and behavioral disorders with the patient prescribed stable doses of Adderall and Risperdal. The patient was non-toxic appearing, and his exam was significant for a left hip that was kept flexed and abducted due to pain with any range of motion. Imaging from the transferring facility included routine radiographs of the hip that were normal, and a contrasted CT of the pelvis obscured by motion artifact but with no acute abnormalities was noted. 

**Figure 1:**
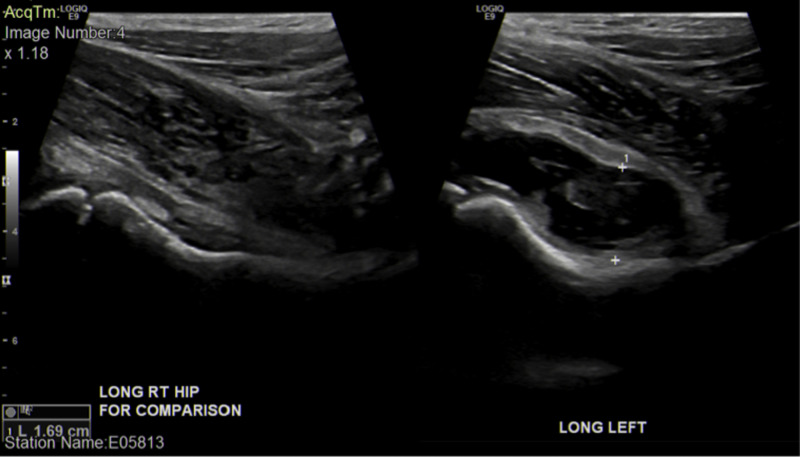
Single long view of the patient’s hips via ultrasound preoperatively. A normal right hip makes the 1.69-cm effusion shown on the left hip more evident. Surrounding hyperemia and loculated effusion versus visualized mass are also seen.

Laboratory workup was significant for an erythrocyte sedimentation rate (ESR) of 16 mm/h (normal <15 mm/h), a C-reactive protein (CRP) of 1.00 mg/dL (normal <0.5 mg/dL), and white blood cell count of 9.91 x 10^3^/microliter without leftward shift. An ultrasound of the hip was obtained, revealing a normal right joint and a 17-mm complex effusion of the left hip with surrounding hyperemia (Figure 1).

The differential diagnosis of acute onset of non-traumatic hip pain in a child eight years of age includes septic arthritis or periarticular infections, such as osteomyelitis or myositis, transient synovitis, and synovitis from Legg-Calve-Perthes disease. Less likely is juvenile idiopathic arthritis (as the hip is very rarely the presenting joint) and synovial-based lesions such as PVNS [1].

**Figure 2:**
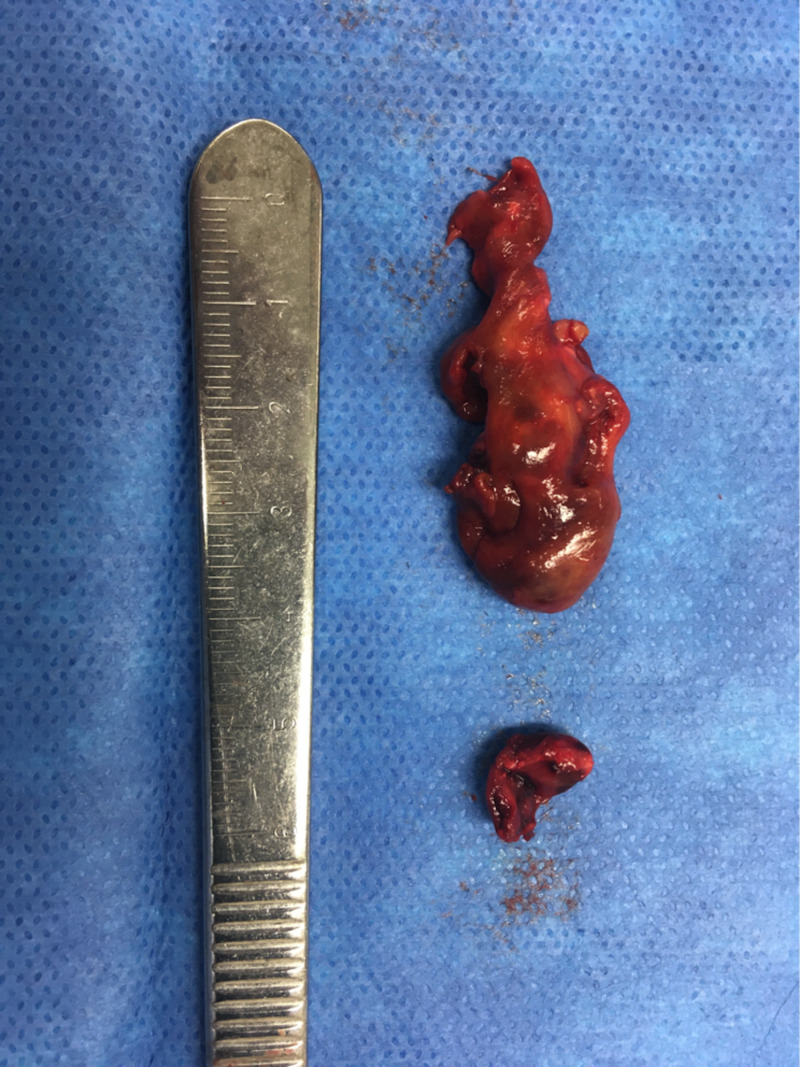
A 4 × 1 × 1.5 cm mass resected form the patients left hip synovium, grossly consistent with pigmented villonodular synovitis.

**Figure 3:**
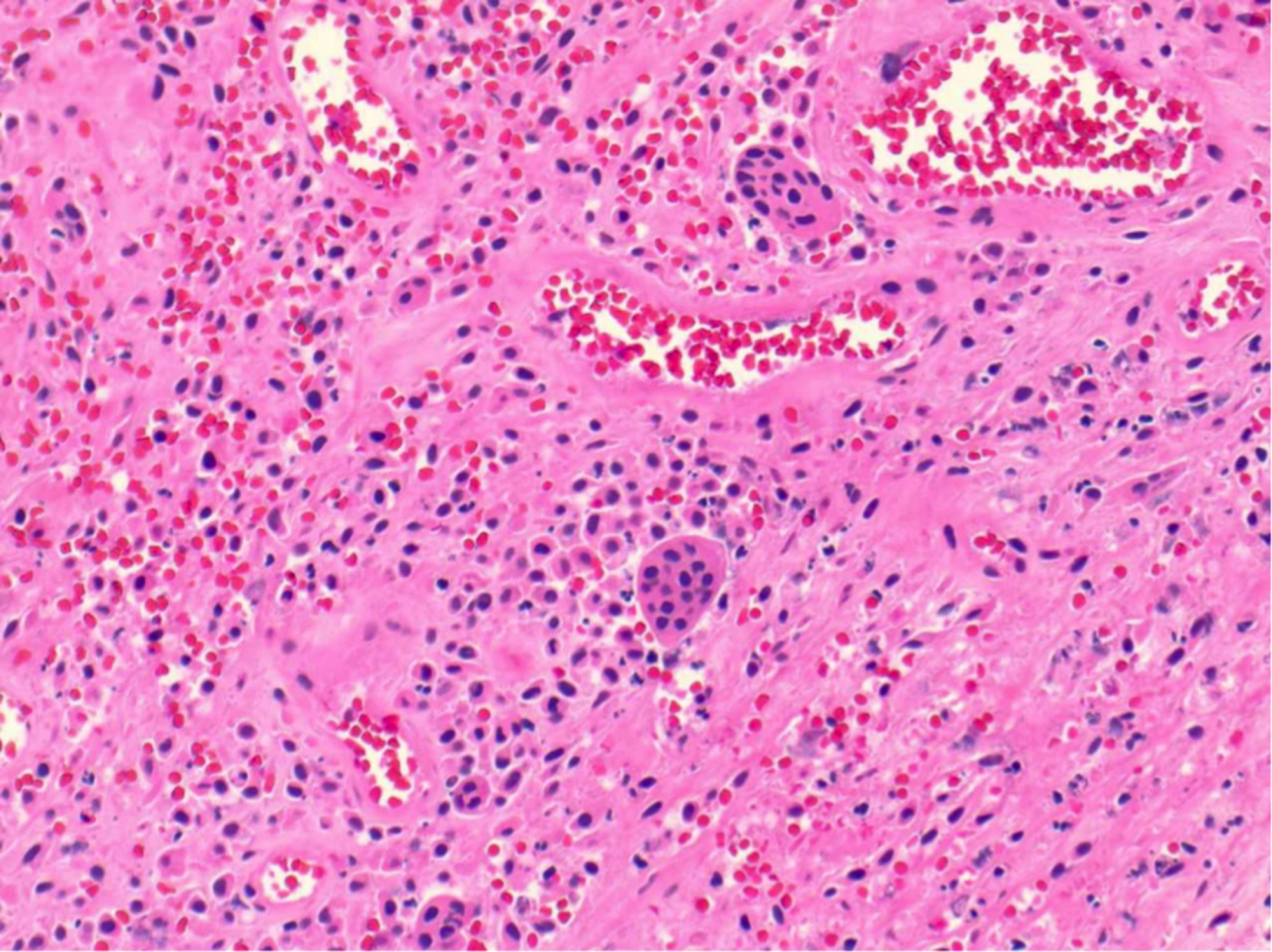
Pathology specimen from the resected mass revealing a lesion primarily with a nodular appearance and a few papillary projections as well plump mononuclear cells in a background of variably collagenized stroma. Occasional hemosiderin-laden macrophages are also noted along with scattered multinucleated giant cells. Morphologic findings are consistent with pigmented villonodular synovitis.

Given the clinical picture, particularly in regard to exquisite pain to range of motion of the hip, the decision was made to proceed with aspiration of the hip under anesthesia, rather than proceeding with further advanced imaging such as MRI, which would have resulted in a delay in treatment. Aspiration was productive of almost eight milliliters of straw colored fluid. At our institution, stat joint fluid analysis after aspiration under anesthesia generally takes between 30 and 45 minutes to return. For this reason, it is not unusual for the surgeon to proceed with formal irrigation and debridement of the joint before joint fluid analysis returns if there is clinical suspicion for septic arthritis. In this case, the decision was made to open the joint based on the range of motion exam and the large effusion. Upon opening the capsule through a medial approach, a large soft tissue mass was seen to be extruding from the joint. It was resected, the joint was irrigated, a drain was placed, and the skin incision closed. The mass was 4 x 1 x 1.5 cm in size (Figure 2). By this time, synovial fluid analysis revealed 900 white blood cells/microliter and a gram stain inconsistent with acute septic arthritis. The patient did well postoperatively and was discharged within 48 hours on oral analgesia. Pathologic analysis of the mass was consistent with PVNS (Figure 3). Further discussion with the patient’s family revealed a similar experience shared by the patient’s father, who had a hip mass discovered and resected in his adolescence (the father’s medical records were not available for review).

## Discussion

When a child presents with acute onset of atraumatic joint pain and on examination has significant pain to joint range of motion, ruling out septic arthritis should be the initial focus. To that end, our patient underwent the appropriate laboratory evaluation for osteoarticular infection, including complete blood count (CBC), ESR, CRP, routine radiography, and ultrasonography. Although the child was afebrile and inflammatory markers were normal, there was still concern about the extreme pain to range of motion and the “complex” effusion noted on ultrasonography. Delay in treatment of septic arthritis, particularly septic arthritis of the hip, can lead to poor outcomes; thus, the decision was made to proceed with immediate aspiration with irrigation and debridement and forego MRI, which would have resulted in a significant delay. Capsular incision with discovery of the mass was both diagnostic and therapeutic. We feel that the current case is important because it adds to the differential diagnosis of acute hip pain in children. It also highlights the challenging nature of obtaining a correct diagnosis in a pediatric patient presenting with acute joint pain, and the importance of multidisciplinary involvement when considering invasive procedures for diagnostic and therapeutic reasons.

PVNS is a rare entity that causes chronic joint pain and limited range of motion. It is less commonly associated with acute arthralgia as we present here. PVNS in pediatric patients is even more rare, with only case reports of occurrence in persons under the age of 18 years [2]. Cases are defined as either diffuse or local relating to the degree of involvement of the joint’s synovium. Symptoms vary but are often more insidious in nature over the course of weeks to months, with time from symptom onset to diagnosis being noted as long as 16 months in one case series [3]. From a pathology standpoint, both types have features consistent with synovial cell proliferation with hemosiderin deposition. Other signs of inflammation, such as lipid-laden macrophages, fibroblast, and stromal cell proliferation, can also be detected [4]. While imaging can raise suspicion for PVNS, tissue is required for formal diagnosis. A multimodal approach for imaging is often taken, with varying benefit from x-ray, CT, and MRI depending on the setting. In terms of diagnostics, MRI is the gold standard for diagnosis, staging, and surveillance [5]. Findings suggestive of PVNS include T1 and T2 hypointensity of the mass, as well as “blooming artifact” resulting in pseudoenlargement of the mass and corresponding with hemosiderin deposits. The differential diagnosis based on imaging alone, primarily from adult literature, details localized synovial hemangioma, hemophilic arthropathy, synovial sarcoma, and chronic tophaceous gout [6]. In our case, MRI was not obtained because of the acute presentation and logistical challenges involved in obtaining an MRI in a reasonable time frame.

Similar cases and presentations are rare in the literature. A case series from a patient population with similar demographics reviewed 17 cases of pediatric PVNS over an 11-year period. In this series, there was an average of 16 months from symptom onset to diagnosis. Only five patients presented with the hip as the affected joint, and of those only one patient experienced acute symptoms of pain and inability to bear weight raising concern for infection and resulting in diagnosis through operative investigation [2]. While the average age of diagnosis is historically in the second to third decade of life, there have been reports of presentation as young as one year of age [7]. At time of presentation, the differential diagnosis focused on delineating acute infectious processes (osteomyelitis, septic joint, inguinal abscess) from musculoskeletal ones (ruptured hip ligament, avascular necrosis of the hip, bursitis vs synovitis due to trauma). While infectious etiologies are the most worrisome due to the need for acute intervention, our patient’s lack of fever, leukocytosis, or inflammatory marker elevation made this a low risk for infection. With his inability to bear weight, he satisfied only one Kocher criteria for septic arthritis of the hip [8]. However, given the size of the effusion, which was noted to be the largest hip effusion encountered by the attending pediatric orthopedic surgeon, the pursuit of incision and drainage ultimately led to the correct diagnosis and treatment. Another interesting point is the patient's family history, with his father reporting a similar mass excised from his hip previously (although his records were unavailable for review). This would point towards a genetic predisposition to developing PVNS, as seen in patients with CSF1 gene mutations responsible for receptor tyrosine kinase expression regulating cell growth [9].

## Conclusions

The patient had immediate improvement in his pain postoperatively and was discharged 48 hours after his procedure with a rolling walker. The family has transportation challenges and has not made their postoperative follow-up appointments, but the mother has been contacted on a number of occasions and she reports complete resolution of his symptoms. He has been scheduled to return for MRI of the hip to evaluate for residual disease. This case recognizes PVNS is an underthought and possibly underdiagnosed cause of joint pain in the pediatric population presumably due to limited growth/size in pediatric patients sufficient enough to result in symptoms. Surgical resection appears to have good outcomes in return of function and lower risk of recurrence in pediatric patients when compared to adults. While presentation and progression of symptoms is typically insidious, PVNS should be in the differential for an acute joint pain without infectious symptoms.
